# Self-Compassion Correlates of Anxiety and Depression Symptoms in Youth: A Comparison of Two Self-Compassion Measures

**DOI:** 10.3390/children9121930

**Published:** 2022-12-08

**Authors:** Peter Muris, Kris Bongers, Claudia Schenning, Cor Meesters, Henry Otgaar

**Affiliations:** Department of Clinical Psychological Science, Faculty of Psychology and Neuroscience, Maastricht University, Minderbroedersberg 4-6, 6211 LK Maastricht, The Netherlands

**Keywords:** self-compassion, anxiety and depression, adolescents, Self-Compassion Scale for Youth (SCS-Y), Sussex-Oxford Compassion for the Self Scale (SOCS-S)

## Abstract

Background and purpose: Self-compassion is considered as a protective psychological construct that would shield against the development of emotional problems. The aim of the present study was to compare the ‘protective nature’ of two measures of self-compassion: the Self-Compassion Scale for Youth (SCS-Y) and the Sussex-Oxford Compassion for the Self Scale (SOCS-S). Methods: Eighty-seven adolescents aged 12 to 18 years completed both self-compassion measures as well as scales of anxiety and depression symptoms. Results: SCS-Y and SOCS-S scores were positively correlated, and for both measures it was generally found that higher levels of self-compassion were associated with lower levels of emotional symptoms. However, the uncompassionate self-responding scales of the SCS-Y correlated positively with anxiety and depression symptoms and hence can better be seen as indices of vulnerability. Regression analyses suggested that a positive attitude toward oneself, as measured by the self-kindness scale of the SCS or its SOCS-S equivalent ‘feeling for the person suffering’ is particularly relevant as a buffer against emotional problems. Conclusion: The protective nature of self-compassion can be established by both measures. Caution is advised with the use of the uncompassionate self-responding scales included in the SCS-Y as they appear to measure vulnerability rather than protection.

## 1. Introduction

Compassion is a construct that evolved from a Buddhist tradition pertaining to an openness to the suffering of others with a commitment to relieve it [1]. Early in the 1990s Western psychology and psychiatry started to gain interest in the concept. Taking a resilience perspective, researchers were especially committed to explore self-directed compassion as a protective factor that shields people against the development of mental health issues in times of adversity and stress and whether the promotion of self-compassion during psychological interventions is helpful to alleviate such problems [2]. During the past decades, evidence has accumulated showing that people with high levels of self-compassion have a better mental health than individuals with low levels of self-compassion [3] and there is also support for the positive effects of self-compassion-enhancing treatments in patients who suffer from various psychopathological conditions [4,5].

The vast majority of studies on self-compassion has been conducted in adult populations, but it is good to note that investigations on the role of this protective factor in youth are steadily increasing. A primary reason for this increase is because adolescence is widely regarded as a period of “storm and stress” [6], making it highly relevant to examine variables that potentially promote resilience during this challenging developmental stage. In general, the results accord well with what has been found in research with adults: higher levels of self-compassion are accompanied by lower levels of psychopathology, with most research focusing on emotional problems such as anxiety and depression [7]. Furthermore, although treatment studies in adolescents are sparser than in adults, some research indicates that youth who suffer from mental health problems profit from a compassion-based intervention [8,9,10].

The currently, most widely used definition of self-compassion has been formulated by Neff [11] who proposed that the construct consists of three key components on bipolar ends: (1) self-kindness, which involves being kind and supportive to oneself rather than reacting harsh and judgmental, (2) common humanity, which pertains to the recognition that difficulties constitute a normal part of human life rather than feeling isolated from other people because of one’s shortcomings and imperfections, and (3) mindfulness, which has to do with the ability of keeping a balanced, rational view on personal suffering rather than becoming fully absorbed and overidentifying with one’s problems. To assess these three components of self-compassion, a self-report questionnaire, the Self-Compassion Scale (SCS), was construed [12], of which a short form (SCS-SF) [13] and an age-appropriate version for youth (SCS-Y) [14] have also been developed. In an attempt to cover the bipolar nature of the self-compassion construct, the SCS and its derivative versions not only consist of the compassionate self-responding (CSR) items that directly maps onto the components of self-kindness, common humanity, and mindfulness, but also contain items that reflect their uncompassionate self-responding (USR) counterparts, namely self-judgment, isolation, and overidentification [12]. For scoring the SCS, ratings on the USR items are reversed and summed with ratings on CSR items to produce a total self-compassion score. A recent review of the research literature showed that the SCS is the most widely used instrument for measuring self-compassion within a context of resilience and protection against mental health problems [15].

Although the basic psychometric qualities of the SCS are good (and this is also true for the shortened and youth versions), several scholars have criticized the validity of the scale [15,16,17]. The main source for this critique pertains to the inclusion of the USR items, which seem to reflect a number of well-known toxic processes that have been shown to be operating in a range of psychopathological problems. More specifically, self-judgment shows similarity with self-criticism [18] and self-punishment [19], isolation resembles loneliness and desolation [20], while over-identification shares prominent features with dysregulated negative thinking as seen in worry and rumination [21]. Scholars have argued that vulnerability is not equivalent to reversed protection [22], and indeed evidence demonstrates that USR is more substantially associated with indices of mental health problems than CSR, resulting in inflated relationships with psychopathology when incorporating such items in the computation of a SCS total score [23].

A series of studies has indicated that this validity issue is not only present in research investigating self-compassion in adults, but also plays a role in studies examining this construct in children and adolescents [24,25]. In a recent investigation, Muris et al. [25] asked two samples of adolescents (*N*s being 106 and 52) to complete the SCS or the SCS-SF as well as scales measuring symptoms of anxiety and depression. The results consistently indicated that CSR on its own explained a relatively small but statistically significant proportion of the variance in anxiety and depression symptoms (i.e., 3–14%). However, the contribution of USR was much larger and the inclusion of this component in the SCS total score raised the percentages of explained variance of both types of symptoms considerably (i.e., with 18–27%). Admittedly, no study can be found that examined this inflation effect of USR in the total self-compassion score as measured by the age-downward version of the SCS (i.e., SCS-Y [14]), but considering the fact that the instrument has the same structure and thus also includes USR items, it is likely that the SCS-Y suffers from the same type of validity problem.

In an attempt to improve the assessment of this potentially interesting protective psychological construct, Strauss et al. [26] reviewed the extant literature to examine various definitions of (self-)compassion with the aim to identify its core features. It was concluded that (self-)compassion essentially consists of five elements: (1) recognizing suffering, (2) understanding the universality of suffering, (3) feeling for the person suffering, (4) tolerating uncomfortable feelings, and (5) motivation to act or acting to alleviate suffering. In line with this notion, Gu et al. [27] developed a new instrument, the Sussex-Oxford Compassion Scales (SOCS) that assesses compassionate responding in terms of these five elements. The SOCS includes one version for measuring compassion towards others and one version for measuring self-compassion. The latter version, the SOCS-S—which was also used in the present study, shows good internal consistency, test–retest reliability, and validity as established by a substantial positive correlation with the shortened version of the SCS [28,29]. Although SOCS-S and SCS both measure the construct of self-compassion, the scales have been developed on the basis of different theoretical assumptions and so it might be interesting to study the relations between both measures in more detail and also to explore their differential links to external constructs.

So far, no research can be found that compared the protective value of self-compassion as measured by both the SCS and the SOCS-S within a context of emotional psychopathology in youth. With this in mind, the present study administered the SCS-Y and the SOCS-S, as well as scales measuring anxiety and depression symptoms in a sample of 87 adolescents aged 12 to 18 years. In this way, we were able to examine (1) the relations between both self-compassion questionnaires and their (sub)scales, (2) the relations between various SCS-Y and SOCS-S (sub)scales and symptom measures of anxiety and depression, and (3) the relative contributions of the SCS-Y and SOCS-S (sub)scales to both symptom measures. We hypothesized that (a) there will be positive correlations between the SCS-Y total score and its CSR (sub)scales and the SOCS-S, whereas negative correlations were expected to be found between the USR (sub)scales of the SCS-Y and the SOCS-S, (b) SCS-Y total self-compassion, CSR (sub)scales of the SCS-Y, and SOCS-S scores will be negatively correlated with symptoms of anxiety and depression, while the USR (sub)scales of the SCS-Y will be positively correlated with such symptoms, and (c) the SCS-Y will be a better predictor of anxiety and depression symptoms than the SOCS-S, but this effect will be mainly carried by the inclusion of the (reversed) USR components which have been demonstrated to be intimately linked with psychopathology [15]. When the USR components of the SCS-Y are discarded, the comparison between both self-compassion instruments will become less biased and have a less predictable outcome. Ultimately, this type of research will advance our knowledge on the assessment of self-compassion, and guide researchers and clinicians to choose the most optimal measure to assess this protective construct in young people.

## 2. Method

### 2.1. Participants

The study was conducted with 87 adolescents who were living in the province of Limburg, in the southeastern part of the Netherlands. There were 61 girls (70.1%) and 26 boys (29.9%) who were all aged between 12 and 18 years (*M* = 15.28 years, *SD* = 0.51). The participants followed lower general secondary education (28.7%), higher general secondary education (25.3%), or pre-university education (46.0%), and were in the first (43.7%), second (24.1%), third (21.8%), or fourth (10.3%) grade. No further information on the socio-economic and ethnic background of the participants was available.

### 2.2. Procedure

Because of the COVID-19 pandemic, recruitment via high schools was not possible and therefore a snowball sampling method (via family members, friends, and colleagues as well as online platforms such as Facebook, LinkedIn, and Nextdoor) was used to recruit participants. Youth were provided with a short information letter describing the purpose of the study and asking them whether they were willing to participate. In case they decided to do so, they were guided via a weblink to the online platform Qualtrics where they were asked to give their informed consent for participation. For youth under the age of 16 permission from their parents was also necessary in order to participate in the current study. Once consent was given online, the participants proceeded with the completion of the set of questionnaires (see below) in Qualtrics. Completion of the survey took approximately 15–20 min.

One-hundred-and-fifty-seven adolescents and their parents initially responded positively to this invitation by signing the informed consent form. However, 70 adolescents had to be discarded because they did not (fully) complete the survey. The remaining participants constituted the final sample of 87 adolescents. An a priori power analysis (with α two-tailed = 0.05 and β = 0.20; https://sample-size.net/correlation-sample-size/, accessed on 1 April 2019) indicated that the sample size needed to detect a correlation of |−0.54| (i.e., the average effect size for the correlation between the self-compassion total score and indices of psychopathology as reported in the meta-analysis by MacBeth & Gumley [3]) was 25. To detect a correlation of |−0.31| (i.e., the average effect size for the correlation between CSR and indices of psychopathology [22]), the sample size should include at least 79 participants. Although there was sufficient power to detect the expected correlations, it should also be noted that the sample size was still limited, thereby hindering solid exploration of gender differences (because of the unfortunate unequal distribution of gender) and age effects (given the quite broad age range and possible developmental differences). Eight gift vouchers (10 euro) were raffled among the participating adolescents as a reward for their participation. Prior to the study ethical approval was obtained from the Ethical Review Committee of Psychology and Neuroscience (ERCPN) at Maastricht University (reference number: Master-192-15-04-2018).

### 2.3. Measures

The *Self-Compassion Scale for Youth* (SCS-Y [14]) is a 17-item self-report questionnaire for measuring self-compassion in terms of Neff’s [11] definition: nine items reflect the CSR components of self-kindness (3 items, e.g., “I am kind and supportive to myself when I’m having a hard time”), common humanity (3 items, e.g., “When I’m sad or unhappy, I remember that other people also feel this way at times”), and mindfulness (3 items, e.g., “When something upsets me, I notice my feelings without getting carried away by them”), while eight items represent the USR components of self-judgment (3 items, e.g., “I get mad at myself for not being better at some things”, isolation (3 items, e.g., “When I feel sad or down, it seems like I’m the only one who feels that way”), and overidentification (2 items, e.g., “When I’m feeling bad or upset, I can’t think of anything else at the time”). Items have to be rated on a five-point Likert scale with 1 = almost never, 2 = not very often, 3 = sometimes, 4 = very often, and 5 = almost always, and are summed to yield CSR and USR (subscale) scores as well as a total self-compassion score (which includes the reversed ratings of USR items). The psychometric properties have been shown to be satisfactory: internal consistency and test–retest stability are good, and apart from the validity issue that has been noted in the introduction, there is also evidence for its convergent and divergent validity [14,30].

The *Sussex-Oxford Compassion for the Self Scale* (SOCS-S [27]) consists of 20 items that measure five key elements of self-compassion: recognizing suffering (4 items, e.g., “I notice when I am feeling distressed”), understanding the universality of suffering (4 items, e.g., “Like me, I know that other people also experience struggles in life”), feeling for the person suffering (4 items; e.g., “When I’m going through a difficult time, I feel kindly towards myself”), tolerating uncomfortable feelings (4 items, e.g., “When I’m upset, I try to stay open to my feelings rather than avoid them”), and acting or being motivated to act to alleviate suffering (4 items, e.g., “When I’m going through a difficult time, I try to look after myself”). Items are scored on a five-point Likert scale ranging from 1 = not at all true to 5 = always true, and combined to yield subscale scores and a self-compassion total score. As noted earlier, the psychometric qualities are good [27,28]. For the use with the younger participants of the present study, some items were simplified by making minor changes in wording, while a number of items were more substantially rephrased (e.g., the item “I understand that feeling upset at times is part of human nature” was changed into “I understand that all people feel angry, scared, or sad from time to time” and “I connect with my own distress without letting it overwhelm me” was altered into “I think about my unpleasant feelings without letting them become too intense”). Because the SOCS-S is an ‘adult’ measure that was used for the first time in younger participants, we conducted an additional pilot study to compare the reliability coefficients of the scale in adolescents (aged 12–18 years; *n* = 106, which included the 87 participants of the current study) and young adults (aged 19–27 years; *n* = 73).

The *Children’s Depression Inventory* (CDI [31]) is a widely employed 27-item self-report scale for measuring cognitive, affective, and behavioral symptoms of depression in youth aged between 7 and 18 years. Items (e.g., “I am sad all the time”, “I do everything wrong”, and “Nothing is fun at all”) have to be rated on a three-point scale with 0 = not true, 1 = somewhat true, and 2 = very true. A total score can be calculated by summing ratings of all items and ranges between 0 (no depression symptoms) to 54 (all depression symptoms clearly present). The CDI has good reliability and validity and this appears true in clinical as well as non-clinical samples of children and adolescents [32,33].

The *Youth Anxiety Measure for DSM-5* (YAM-5 [34]) is a questionnaire to assess symptoms of anxiety disorders in terms of the contemporary psychiatric classification system. The YAM-5 consists of two parts: one part measuring symptoms of the major anxiety disorders and another part measuring symptoms of specific phobias. In the present study, we only used the part focusing on the major anxiety disorders (i.e., social anxiety, separation anxiety, generalized anxiety, panic, and selective mutism), which includes 28 items (e.g., “I find it very scary to talk with people I don’t know”, “I am afraid to go anywhere without my parents”, “I worry a lot”, “I panic for no reason”, and “At school I don’t dare to talk to the teacher”) that have to be rated on a four-point Likert scale with 0 = never, 1 = sometimes, 2 = often, and 3 = always. Rather than calculating separate scores for each anxiety disorder, we computed a YAM-5 total score that could possibly range between 0 and 84, with high higher scores indicating higher levels of anxiety symptoms. There is good evidence for the psychometric properties of the measure [35,36,37].

### 2.4. Statistical Analyses

Data were analyzed with the Statistical Package for the Social Sciences (SPSS, version 25). To begin with, descriptive statistics (mean scores, standard deviations, and reliability coefficients) were computed for various questionnaires, and gender differences and age effects were explored by means of independent samples *t*-tests and Pearson correlations. Next, correlations were computed to examine relations between the SCS-Y and the SOCS-S, and between these scales and symptoms of anxiety and depression. Finally, regression analyses were conducted to explore unique contributions of various SCS-Y and SOCS-S scales to both types of emotional symptoms. In model 1, we tested the relative contributions of the SCS-Y and SOCS-S total scores. In model 2, we examined the contributions of SCS-Y CSR and USR separately as well as the SOCS-S total score. In model 3, SCS-Y CSR and the SOCS-S total score were the predictor variables in order to investigate the independent contributions of pure protective scales. Model 4 was a further exploration of model 3: here we looked at a subscale level to find out which self-compassion components were mainly responsible for the ‘protective’ effects. The robustness of the findings of the regression models was tested by means of bootstrapping analyses (3000 samples) to estimate the Bias-Corrected and accelerated 95% Confidence Interval (BCa 95% CI). To evaluate whether multicollinearity was an issue in the regression models, the Variance Inflaction factor (VIF) was calculated for predictor variables. For models 1 to 3, VIF values of all predictor variables were all between 1.90 and 2.62, indicating that multicollinearity was not a significant problem. In model 4, two SOCS-S subscales (i.e., ‘feeling for the person suffering’ and ‘acting to alleviate suffering’) displayed somewhat higher VIF statistics (i.e., between 6.34 and 7.74), which pointed out that these subscales were more strongly related to other variables, but these values were still within an acceptable range (i.e., VIF < 10) [38].

## 3. Results

### 3.1. General Findings

Before addressing the main research questions of this study, a number of general findings should be discussed. First, *t*-tests revealed that there were statistically significant gender differences on a number of the scales (Table 1), although these need to be interpreted with caution given the unequal distribution of boys and girls in the present sample. More precisely, on the SCS-Y, girls reported higher levels of USR than boys [*t*(85) = 2.37, *p* = 0.02], and this difference was mainly carried by the subscales of self-judgment [*t*(85) = 2.41, *p* = 0.02] and overidentification [*t*(85) = 2.07, *p* = 0.04]. On the SOCS-S, only one significant gender difference was found: girls displayed higher levels of ‘understanding the universality of suffering’ than boys [*t*(34.56) = 2.19, *p* = 0.04]. On the CDI and YAM-5, it was found that girls exhibited higher symptom levels than boys [depression: *t*(85) = 2.31, *p* = 0.02; anxiety: *t*(85) = 2.33, *p* = 0.02]. Second, age did not have a significant influence on any of the measured variables. That is, correlations between age and youth’s questionnaire scores were small and non-significant. Third, the internal consistency coefficients of various questionnaires that were used in this study were generally satisfactory (i.e., Cronbach’s alphas > 0.70). There were only two notable exceptions: for SCS overidentification and SOCS-S ‘tolerating uncomfortable feelings’, lower alpha values of 0.63 and 0.68 were found, which was not that surprising given the limited number of items in both subscales (i.e., 2 and 4, respectively).

The results of the pilot study, which was conducted to compare the reliability of the SOCS-S in adolescents versus young adults, revealed that the internal consistency coefficients of younger and older persons were highly comparable. Cronbach’s alphas were, respectively, 0.93 and 0.90 for the SOCS-S total score, 0.74 and 0.72 for ‘recognizing suffering’, 0.89 and 0.78 for ‘understanding the universality of suffering’, 0.85 and 0.86 for ‘feeling for the person suffering’, 0.70 and 0.63 for ‘tolerating uncomfortable feelings’, and 0.86 and 0.85 for ‘acting to alleviate suffering’. This indicates that the SOCS-S can be used to obtain reliable estimates of self-compassion in adolescents.

### 3.2. Correlations between Both Self-Compassion Scales

Table 2 displays partial correlations (corrected for gender) between the SCS-Y and the SOCS-S. As can be seen, the total scores of both self-compassion scales were substantially and positively correlated (*r* = 0.78, *p* = 0.000). Further, the CSR components of the SCS-Y correlated positively and statistically significantly with most SOCS-S subscales, while the USR components of the SCS-Y correlated negatively with the majority of SOCS-S subscales. Particularly strong correlations were noted between the SCS-Y total, CSR, and self-kindness scores and the SOCS-S total score and the subscales ‘feeling for the person suffering’, ‘tolerating uncomfortable feelings’, and ‘alleviating suffering’ (all *r*s between 0.64 and 0.82, *p*s = 0.000). Small and mostly non-significant correlations were noted between the SCS-Y and the SOCS-S recognizing suffering. Finally, we had special interest in correlations between subscales in both measures that intend to measure the same aspect of self-compassion. It was found that SCS-Y self-kindness and SOCS-S feeling for the person suffering were strongly correlated (*r* = 0.80, *p* = 0.000), while the partial correlation between SCS-Y common humanity and SOCS-S understanding the universality of suffering was more modest (*r* = 0.42, *p* = 0.000).

### 3.3. Correlations between Self-Compassion and Symptoms of Anxiety and Depression

Partial correlations (corrected for gender) between both self-compassion scales and symptom measures of anxiety and depression are shown in Table 3. As can be seen, the results were as hypothesized. That is, the total self-compassion score and the CSR (sub)scales of the SCS-Y were negatively correlated with symptoms of anxiety and depression, while the USR (sub)scales of the SCS-Y were positively correlated with such symptoms. The only finding that deviated from this general pattern involved the common humanity subscale of the SCS-Y, which did not show a statistically significant negative correlation with anxiety symptoms (*r* = −0.13, *p* = 0.25). Further, the SOCS-S scales were generally negatively correlated with symptoms of anxiety and depression. There was again one exception: the subscale ‘recognizing suffering’ was not statistically significantly associated with depression symptoms (*r* = −0.18, *p* = 0.11).

### 3.4. Relative Contributions of Both Self-Compassion Measures to Symptoms of Anxiety and Depression

Regression analyses (corrected for gender by entering this demographic variable in the models on step 0) were carried out to examine the unique contributions of SCS-Y and SOCS-S scales to both types of emotional symptoms. The results for the four tested models are displayed in Table 4 and Table 5. As can be seen, across various models, both self-compassion scales explained between 24–32% and 33–44% of the variance in, respectively, anxiety and depression symptoms.

In model 1, the SCS-Y and SOCS-S total scores were the predictor variables. As hypothesized, the SCS-Y total score was found to be a significant negative predictor of both anxiety and depression symptoms (βs being −0.31, *p* = 0.02 and −0.53, *p* = 0.000), a result that was supported by the BCa 95% CI’s of [−0.75, −0.07] and [−0.65, −0.25], which did not include ‘0’. Note that in this model the SOCS-S total score did not make a statistically significant contribution.

For model 2, the SCS-Y was split into its CSR and USR components and these were entered as predictors in the regression equation along with the SOCS-S. The results showed that it was especially the SCS-Y USR component that made a unique positive contribution to symptoms of anxiety and depression (βs being 0.40 and 0.37, both *p*s = 0.000; BCa 95% CI’s being [0.44, 1.35] and [0.23, 0.92], respectively). In this model, the CSR component of the SCS-Y did not emerge as a significant predictor of symptoms, but the SOCS-S was found to make a significant independent contribution in the case of anxiety symptoms (β = −0.29, *p* = 0.04). Note, however, that the bootstrapping procedure yielded a BCa 95% CI that just included 0 and thus question the robustness of this finding.

Model 3 included SCS-Y CSR and the SOCS-S total score as predictor variables to explore the relative contributions of these pure protective scales to both types of emotional symptoms. In the case of anxiety symptoms, only SOCS-S made a significant unique contribution (β = −0.49, *p* = 0.000; BCa 95% CI = [−0.74, −0.21]), whereas in the case of depression symptoms SOCS-S and SCS-Y both explained significant proportions of the variance (βs being −0.33, *p* = 0.01 and −0.29, *p* = 0.02; BCa 95% CI’s being [−0.38, −0.10] and [−0.74, −0.08], respectively). To further explore these results, model 4 examined at a subscale level which self-compassion components were mainly responsible for the ‘protective’ effects noted in model 3. In the regression analysis predicting anxiety symptoms, SOCS-S ‘feeling for the person suffering’ appeared to be the only significant predictor (β = −0.46, *p* = 0.04, although the result should be interpreted with prudence given BCa 95% CI = [−3.63, 0.09]). In the regression analysis predicting depression symptoms, SCS-Y self-kindness was the only variable making a unique and significant contribution (β = −0.47, *p* = 0.003; BCa 95% CI = [−2.99, −0.35]).

## 4. Discussion

The present study examined self-compassion correlates of anxiety and depression symptoms in a sample of adolescents who were recruited in the general population by means of snowball sampling. Two measures were used to measure self-compassion: an age-downward version of Neff’s [12] widely employed SCS (i.e., SCS-Y [14]) and the more recently developed SOCS-S [27]. The SCS-Y is based on Neff’s [11] conceptualization which defines self-compassion as an equilibrium of the CSR components of self-kindness, common humanity, and mindfulness and their USR counterparts of self-judgment, isolation, and overidentification. The SOCS-S was construed following a comprehensive review of the (self-)compassion literature and contains all the relevant elements that according to various theories and definitions were considered relevant for this psychological construct [26]. Although it should be acknowledged that the SOCS-S had not been used before in adolescent populations, we thought that with some adjustments the scale would certainly be useful in younger people and provide an alternative measure of self-compassion that we could compare to the SCS.

The results first of all showed that both measures of self-compassion were substantially correlated [29], and this appeared not only true for the total scores of both questionnaires but also for various (sub)scales. The one clear exception involved the SOCS-S subscale ‘recognizing suffering’, which displayed rather weak correlations with various SCS-Y (sub)scales. This subscale primarily measures to what extent a person is capable of perceiving signs of distress in him/herself, which can better be regarded as a prerequisite for showing the prototypical affective, cognitive, and behavioral signs of a compassionate self-response [26]. Obviously, this element is not explicitly covered by the SCS-Y [14].

A second finding was that both self-compassion scales were negatively correlated with symptoms of anxiety and depression. This indicates that higher levels of self-compassion as measured with both scales are accompanied with lower levels of emotional symptoms, which is in line with what has been noted in previous research with both adults and younger people [2,3,7] and can be taken as support for the ‘protective’ nature of this psychological construct [15]. At a subscale level, the SCS-Y CSR components of self-kindness, common humanity, and mindfulness correlated negatively with symptoms of anxiety and depression, while the SCS-Y USR components of self-judgment, isolation, and overidentification correlated positively with such emotional symptoms. This has led some scholars to the conclusion that the former indeed reflect protective features of the self-compassion construct, while the latter can better be seen as indices of increased vulnerability [22].

There were three subscales that did not show (the expected) substantial negative correlations with the symptom scales. These were the common humanity subscale of the SCS-Y (which correlated only modestly with symptoms of depression), the ‘recognizing suffering’ subscale of the SOCS-S (which only showed a small but statistically significant correlation with anxiety symptoms), and the ‘understanding the universality of suffering’ subscale of the SOCS-S (which displayed modest but statistically significant correlations with both symptom measures). These three subscales do represent features of self-compassion that are related to positive and negative life outcomes [26,39], but they seem to be less coping-oriented than other self-compassion elements, which could explain why they have a more limited impact on the experience and modulation of emotional symptoms. The need for investigating the relationship between various self-compassion components and coping has been signaled as an important topic for future inquiry [40].

The third goal of this study was to examine the relative contributions of both self-compassion measures to symptoms of anxiety and depression. This was done by means of regression analyses and the findings can be summarized in two main conclusions. To begin with, the data are a further illustration of the previously made point that the inclusion of the USR components in the SCS(-Y) (a) will inflate the (negative) relation between self-compassion and emotional symptoms and hence unjustly boost the protective power of this construct, and (b) will blur the comparison of self-compassion with other psychological constructs of protection or vulnerability that are relevant within a context of psychopathology (in this case: self-compassion as measured with an alternative scale; see [25]). Given the accumulating, univocal evidence in support of this notion, the obvious recommendation for researchers would be to discard the USR items when using the SCS or its shortened and down-aged equivalents when investigating the protective trait of self-compassion. The second conclusion is that the data suggest that some components/elements of self-compassion are more important for understanding emotional symptoms such as anxiety and depression than others. More specifically, the present findings suggest that a positive, proactive attitude toward oneself, as measured by the self-kindness scale of the SCS or its SOCS-S equivalent ‘feeling for the person suffering’, is a particularly relevant element of the self-compassion construct when studying its protective role for these types of psychopathology. Some scholars have argued that it is worthwhile to particularly focus on this defining feature of self-compassion to learn more about its unique role in people’s resilience against mental health issues [41]. Meanwhile, it is good to bear in mind that compassion-based interventions often target other elements than self-kindness (e.g., mindfulness [42]), and there is some evidence that the enhancement of other components (e.g., mindfulness and common humanity) is needed to promote a more positive attitude toward oneself [43].

The present study also yielded a number of additional findings that warrant brief discussion. First, statistically significant gender differences were found for some measures. Girls displayed higher levels of anxiety and depression symptoms and USR—in particular its components of isolation and overidentification, which is in keeping with previous research showing that adolescent females generally report higher levels of internalizing psychopathology [44,45] and associated features of emotional vulnerability. On indices of ‘true’ self-compassion, scores were mostly comparable between both genders, with the one exception of girls scoring higher on SOCS-S ‘understanding the universality of suffering’. This result is not in line with the observation of some scholars that males tend to display higher levels of self-compassion than females [46]. Note, however, that many studies have relied on the SCS or SCS-SF total score and that a closer inspection has revealed that this gender effect is mainly carried by the fact that males score lower on the USR components of the scale. Second, the SCS-Y and the SOCS-S are relatively new measures of which the basic psychometric properties have not been extensively tested in previous research. That is, the SCS-Y was only recently developed and evaluated [14,30], and the same is true for the SOCS-S which has also been investigated in a limited number of studies [27,28,29]. The present study indicates that the concurrent validity is good and that the internal consistency of both scales seems to be acceptable, although reliability estimates of some subscales might be suppressed due to a small number of items.

It should be acknowledged that this study suffers from a number of limitations. To begin with, the study relied on a rather small sample of adolescents who were recruited by means of a snowball sampling method. This method was used because our standard recruitment via the schools was not possible due to COVID-19 restrictions, but we need to be aware of the fact that this type of approach is susceptible to biases that may threaten the generalizability of the findings. A related shortcoming was that the sample included more girls than boys, which hindered us in a thorough examination of gender effects. A further drawback pertains to the correlational design of the study. Although perfectly suitable for testing the relations between both self-compassion measures, such a set-up is less appropriate for testing the actual protective role of self-compassion within a context of emotional psychopathology as no conclusions regarding cause-effect relations can be drawn (see [15]). Another demerit concerns the fact that this was the first study that used the SOCS-S in an adolescent sample. Although the results indicated that internal consistency reliability was good (and comparable to what has been found in adult samples) and the correlations with the SCS-Y provide some support for its concurrent validity, the scale has so far not been subjected to a thorough psychometric evaluation in younger people and so this is obviously a topic of further inquiry. A final limitation was that the adolescents in this study were recruited in the community and that most of them displayed low to moderate levels of anxiety and depression symptoms. To establish the protective features of self-compassion, it seems particularly important to examine the relations with symptom levels in clinically referred youth who suffer from anxiety and depressive disorders.

## 5. Conclusions

The results of the current study indicate that the SCS-Y (and especially its CSR components) and the SOCS-S both appear to be reliable and valid instruments for measuring self-compassion in young people. Thus, both scales seem to be useful for researchers who aim to investigate the protective influence of this individual difference variable within a context of emotional psychopathology, or clinicians who want to monitor progress in youth’s self-directed attitudes of kindness and acceptance during the course of psychological treatment. Meanwhile, cautionary note should be made regarding the use of the USR scales included in the SCS-Y as these appear to measure vulnerability rather than protection. Interestingly, some indications were found that a positive attitude toward oneself, as measured by the self-kindness scale of the SCS or its SOCS-S equivalent ‘feeling for the person suffering’, seems to be a particularly important element of self-compassion as a buffer against symptoms of anxiety and depression. Maybe this finding is not that surprising given that self-kindness, of all self-compassion components, seems to reflect most closely the affiliative orientation to oneself, which seems to be the prototypical feature of this protective construct [41]. For this reason, promotion of self-kindness can be considered as a crucial target in compassion-focused treatment of mental health problems [47]. Obviously, more studies are warranted that focuses on the relative contributions of various self-compassion components/elements in emotional psychopathology and their importance during psychological treatments. Ultimately, such research could also lead to further improvements of interventions for youth with anxiety and depressive disorders.

## Figures and Tables

**Table 1:** General statistics (means, standard deviations, gender differences, and reliability coefficients) of various questionnaires used in this study.

	Total Sample (*n* = 87)	Boys (*n* = 26)	Girls (*n* = 61)	Cronbach’s α
SCS-Y				
Total self-compassion ^†^	50.10 (11.28)	52.85 (11.85)	48.93 (10.93)	0.90
CSR	26.95 (6.40)	27.27 (6.52)	26.82 (6.40)	0.86
Self-kindness	9.22 (2.47)	9.38 (2.30)	9.15 (2.55)	0.77
Common humanity	8.40 (2.60)	8.35 (3.03)	8.43 (2.42)	0.76
Mindfulness	9.33 (2.49)	9.54 (2.27)	9.25 (2.59)	0.71
USR	24.85 (6.40)	22.42 (7.06)	25.89 (5.86) *	0.86
Self-judgment	9.37 (3.05)	8.19 (3.43)	9.87 (2.75) *	0.81
Isolation	8.72 (2.69)	8.08 (2.80)	9.00 (2.61)	0.71
Overidentification	6.76 (1.81)	6.15 (1.85)	7.02 (1.75) *	0.63
SOCS-S				
Total self-compassion	68.33 (13.60)	65.54 (16.60)	69.52 (12.07)	0.93
Recognizing suffering	15.51 (2.75)	14.58 (3.25)	15.90 (2.43)	0.75
Understanding universality suffering	15.54 (3.47)	14.11 (4.35)	16.15 (2.86) *	0.89
Feeling for the person suffering	12.57 (3.54)	12.81 (3.70)	12.48 (3.49)	0.85
Tolerating uncomfortable feelings	11.99 (3.02)	11.73 (3.42)	12.10 (2.86)	0.68
Acting to alleviate suffering	12.72 (3.70)	12.31 (3.88)	12.90 (3.64)	0.89
CDI				
Depression	12.06 (9.42)	8.58 (8.28)	13.54 (9.55) *	0.92
YAM-5				
Anxiety	18.86 (13.52)	13.81 (10.40)	21.02 (14.19) *	0.93

*Note*. SCS-Y = Self-Compassion Scale for Youth, CSR = Compassionate Self-Responding, USR = Uncompassionate Self-Responding, SOCS-S = Sussex-Oxford Compassion for the Self Scale, CDI = Children’s Depression Inventory, YAM-5 = Youth Anxiety Measure for DSM-5. ^†^ Including reversed negative (i.e., USR) items. * Significant gender difference at *p* < 0.05.

**Table 2:** Partial correlations (corrected for gender) between the SCS-Y and SOCS-S.

	1	2	3	4	5	6	7	8	9	10	11	12	13	14
SCS-Y														
1 Total self-compassion ^†^														
2 CSR	0.89 **													
3 Self-kindness	0.82 **	0.86 **												
4 Common humanity	0.71 **	0.82 **	0.54 **											
5 Mindfulness	0.74 **	0.86 **	0.67 **	0.54 **										
6 USR	−0.88 **	−0.56 **	−0.59 **	−0.42 **	−0.43 **									
7 Self-judgment	−0.73 **	−0.43 **	−0.45 **	−0.34 *	−0.30 *	0.87 **								
8 Isolation	−0.72 **	−0.43 **	−0.47 **	−0.32 *	−0.32 *	0.85 **	0.56 **							
9 Overidentification	−0.79 **	−0.61 **	−0.60 **	−0.45 **	−0.52 **	0.78 **	0.54 **	0.57 **						
SOCS-S														
10 Total self-compassion	0.78 **	0.73 **	0.69 **	0.56 **	0.61 **	−0.65 **	−0.57 **	−0.46 **	−0.64 **					
11 Recognizing suffering	0.23 *	0.21	0.19	0.21	0.14	−0.20	−0.25 *	−0.03	−0.24 *	0.64 **				
12 Understanding universality suffering	0.52 **	0.46 **	0.34 *	0.42 **	0.41 **	−0.46 **	−0.42 **	−0.33 *	−0.44 **	0.80 **	0.60 **			
13 Feeling for person suffering	0.81 **	0.75 **	0.80 **	0.51 **	0.61 **	−0.69 **	−0.55 **	−0.54 **	−0.70 **	0.88 **	0.36 *	0.55 **		
14 Tolerating uncomfortable feelings	0.74 **	0.72 **	0.64 **	0.59 **	0.61 **	−0.60 **	−0.52 **	−0.46 **	−0.51 **	0.87 **	0.41 **	0.61 **	0.75 **	
15 Alleviating suffering	0.82 **	0.77 **	0.78 **	0.53 **	0.66 **	−0.67 **	−0.57 **	−0.48 **	−0.68 **	0.90 **	0.39 **	0.56 **	0.92 **	0.78 **

*Note*. *n* = 87. SCS-Y = Self-Compassion Scale for Youth, CSR = Compassionate Self-Responding, USR = Uncompassionate Self-Responding, SOCS-S = Sussex-Oxford Compassion for the Self Scale. ^†^ Including reversed negative (i.e., USR) items. * *p* < 0.05, ** *p* < 0.001.

**Table 3:** Partial correlations (corrected for gender) between both self-compassion measures (SCS-Y and SOCS-S) and depression and anxiety scales.

	YAM-5 Anxiety	CDI Depression
SCS-Y		
Total self-compassion ^†^	−0.52 **	−0.65 **
CSR	−0.36 *	−0.54 **
Self-kindness	−0.44 **	−0.65 **
Common humanity	−0.13	−0.29 *
Mindfulness	−0.37 **	−0.46 **
USR	0.55 **	0.60 **
Self-judgment	0.45 **	0.52 **
Isolation	0.45 **	0.46 **
Overidentification	0.52 **	0.52 **
SOCS-S		
Total self-compassion	−0.50 **	−0.55 **
Recognizing suffering	−0.30 *	−0.18
Understanding universality suffering	−0.34 *	−0.32 *
Feeling for the person suffering	−0.54 **	−0.61 **
Tolerating uncomfortable feelings	−0.35 *	−0.49 **
Acting to alleviate suffering	−0.51 **	−0.61 **

*Note*. *n* = 87. SCS-Y = Self-Compassion Scale for Youth, CSR = Compassionate Self-Responding, USR = Uncompassionate Self-Responding, SOCS-S = Sussex-Oxford Compassion for the Self Scale, YAM-5 = Youth Anxiety Measure for DSM-5, CDI = Children’s Depression Inventory. ^†^ Including reversed negative (i.e., USR) items. * *p* < 0.05, ** *p* < 0.001.

**Table 4:** Results of the linear regression analyses predicting **anxiety symptoms** from various compassion measures (SCS-Y and SOCS-S).

	YAM-5 Anxiety			
	B [BCa 95%CI]	SE	β	*R* ^2^
Model 1				0.28 **
SCS-Y Total self-compassion ^†^	−0.37 [−0.75, −0.07]	0.17	−0.31 *	
SOCS-S Total self-compassion	−0.25 [−0.52, 0.09]	0.14	−0.25	
Model 2				0.32 **
SCS-Y CSR	0.17 [−0.48, 0.75]	0.27	0.08	
SCS-Y USR	0.85 [0.44, 10.35]	0.25	0.40 **	
SOCS-S Total self-compassion	−0.29 [−0.58, 0.04]	0.14	−0.29 *	
Model 3				0.24 **
SCS-Y CSR	0.00 [−0.64, 0.57]	0.28	0.00	
SOCS-S Total self-compassion	−0.49 [−0.74, −0.21]	0.13	−0.49 **	
Model 4 ^#^				0.30 **
SCS-Y Self-kindness SCS-Y Common humanity				
SCS-Y Mindfulness				
SOCS-S Recognizing suffering	−0.69 [−1.92, 0.39]	0.57	−0.14	
SOCS-S Understanding universality suffering	−0.09 [−1.27, 10.24]	0.53	−0.02	
SOCS-S Feeling for the person suffering SOCS-S Tolerating uncomfortable feelings SOCS-S Acting to alleviate suffering	−10.77 [−3.63, 0.09] 0.96 [−0.15, 1.96] −0.63 [−2.37, 1.11]	0.86 0.68 0.86	−0.46 * 0.21 −0.17	

*Note*. *N* = 87. All analyses were corrected for gender by entering this variable in the regression model on step 0. SCS-Y = Self-Compassion Scale for Youth, CSR = Compassionate Self-Responding, USR = Uncompassionate Self-Responding, SOCS-S = Sussex-Oxford Compassion for the Self Scale, YAM-5 = Youth Anxiety Measure for DSM-5. BCa 95% CI = Bias-Corrected and accelerated 95% Confidence Interval (bootstrapping 3000 samples). ^†^ Including reversed negative (i.e., USR) items. ^#^ Only subscales of self-compassion measures that contributed significantly to model 3 were included in this analysis. * *p* < 0.05, ** *p* < 0.001.

**Table 5:** Results of the linear regression analyses predicting **depression symptoms** from various compassion measures (SCS-Y and SOCS-S).

	CDI Depression			
	B [BCa 95%CI]	SE	β	*R* ^2^
Model 1				0.40 **
SCS-Y Total self-compassion ^†^	−0.44 [−0.65, −0.25]	0.11	−0.53 **	
SOCS-S Total self-compassion	−0.09 [−0.25, 0.08]	0.09	−0.13	
Model 2				0.40 **
SCS-Y CSR	−0.33 [−0.60, 0.00]	0.18	−0.22	
SCS-Y USR	0.55 [0.23, 0.92]	0.17	0.37 **	
SOCS-S Total self-compassion	−0.10 [−0.27, 0.07]	0.09	−0.14	
Model 3				0.33 **
SCS-Y CSR	−0.43 [−0.74, −0.08]	0.18	−0.29 *	
SOCS-S Total self-compassion	−0.23 [−0.38, −0.10]	0.09	−0.33 *	
Model 4 ^#^				0.44 **
SCS-Y Self-kindness SCS-Y Common humanity	−1.78 [−2.99, −0.35] 0.56 [−0.22, 1.21]	0.58 0.38	−0.47 * 0.16	
SCS-Y Mindfulness	0.04 [−0.84, 0.98]	0.46	0.01	
SOCS-S Recognizing suffering	0.18 [−0.47, 0.93]	0.37	0.05	
SOCS-S Understanding universality uffering	−0.23 [−0.94, 0.61]	0.35	−0.09	
SOCS-S Feeling for the person suffering SOCS-S Tolerating uncomfortable feelings SOCS-S Acting to alleviate suffering	−0.22 [−1.41, 1.00] −0.13 [−0.92, 0.67] −0.48 [−1.59, 0.47]	0.58 0.45 0.57	−0.08 −0.04 −0.19	

*Note*. *n* = 87. All analyses were corrected for gender by entering this variable in the regression model on step 0. SCS-Y = Self-Compassion Scale for Youth, CSR = Compassionate Self-Responding, USR = Uncompassionate Self-Responding, SOCS-S = Sussex-Oxford Compassion for the Self Scale, CDI = Children’s Depression Inventory. BCa 95% CI = Bias-Corrected and accelerated 95% Confidence Interval (bootstrapping 3000 samples). ^†^ Including reversed negative (i.e., USR) items. ^#^ Only subscales of self-compassion measures that contributed significantly to model 3 were included in this analysis. * *p* < 0.05, ** *p* < 0.001.

## Data Availability

Data can be obtained on request from the corresponding author.

## References

[1] Lama D. (2001). An Open Heart: Practicing Compassion in Everyday Life.

[2] Barnard L.K., Curry J.F. (2011). Self-Compassion: Conceptualizations, Correlates, & Interventions. Rev. Gen. Psychol..

[3] MacBeth A., Gumley A. (2012). Exploring compassion: A meta-analysis of the association between self-compassion and psychopathology. Clin. Psychol. Rev..

[4] Ferrari M., Hunt C., Harrysunker A., Abbott M.J., Beath A.P., Einstein D.A. (2019). Self-Compassion Interventions and Psychosocial Outcomes: A Meta-Analysis of RCTs. Mindfulness.

[5] Gilbert P. (2020). Compassion: From Its Evolution to a Psychotherapy. Front. Psychol..

[6] Hall G.S. (1904). Adolescence: In Psychology and ITS relation to Physiology, Anthropology, Sociology, Sex, Crime, Religion, and Education.

[7] Marsh I.C., Chan S.W.Y., MacBeth A. (2018). Self-compassion and Psychological Distress in Adolescents—A Meta-analysis. Mindfulness.

[8] Bluth K., Gaylord S.A., Campo R.A., Mullarkey M.C., Hobbs L. (2016). Making Friends with Yourself: A Mixed Methods Pilot Study of a Mindful Self-Compassion Program for Adolescents. Mindfulness.

[9] Boggiss A.L., Consedine N.S., Schache K.R., Jefferies C., Bluth K., Hofman P.L., Serlachius A.S. (2020). A brief self-compassion intervention for adolescents with type 1 diabetes and disordered eating: A feasibility study. Diabet. Med..

[10] Donovan E., Rodgers R.F., Cousineau T.M., McGowan K.M., Luk S., Yates K., Franko D.L. (2016). Brief report: Feasibility of a mindfulness and self-compassion based mobile intervention for adolescents. J. Adolesc..

[11] Neff K. (2003). Self-Compassion: An Alternative Conceptualization of a Healthy Attitude Toward Oneself. Self Identity.

[12] Neff K.D. (2003). The Development and Validation of a Scale to Measure Self-Compassion. Self Identity.

[13] Raes F., Pommier E., Neff K.D., Van Gucht D. (2011). Construction and factorial validation of a short form of the Self-Compassion Scale. Clin. Psychol. Psychother..

[14] Neff K.D., Bluth K., Tóth-Király I., Davidson O., Knox M.C., Williamson Z., Costigan A. (2021). Development and Validation of the Self-Compassion Scale for Youth. J. Pers. Assess..

[15] Muris P., Otgaar H. (2020). The Process of Science: A Critical Evaluation of more than 15 Years of Research on Self-Compassion with the Self-Compassion Scale. Mindfulness.

[16] Brenner R.E., Vogel D.L., Lannin D.G., Engel K.E., Seidman A.J., Heath P.J. (2018). Do self-compassion and self-coldness distinctly relate to distress and well-being? A theoretical model of self-relating. J. Couns. Psychol..

[17] Lopez A., Sanderman R., Smink A., Zhang Y., Van Sonderen E., Ranchor A., Schroevers M.J. (2015). A reconsideration of the Self-Compassion Scale’s total score. PLoS ONE.

[18] Werner A.M., Tibubos A.N., Rohrmann S., Reiss N. (2019). The clinical trait self-criticism and its relation to psychopathology: A systematic review—Update. J. Affect. Disord..

[19] de Vel-Palumbo M., Woodyatt L., Wenzel M. (2018). Why do we self-punish? Perceptions of the motives and impact of self-punishment outside the laboratory. Eur. J. Soc. Psychol..

[20] Ernst J.M., Cacioppo J.T. (1999). Lonely hearts: Psychological perspectives on loneliness. Appl. Prev. Psychol..

[21] Watkins E.R. (2008). Constructive and unconstructive repetitive thought. Psychol. Bull..

[22] Muris P., Petrocchi N. (2016). Protection or Vulnerability? A Meta-Analysis of the Relations Between the Positive and Negative Components of Self-Compassion and Psychopathology. Clin. Psychol. Psychother..

[23] Neff K.D. (2023). Self-Compassion: Theory, Method, Research, and Intervention. Annu. Rev. Psychol..

[24] Muris P., Broek M.V.D., Otgaar H., Oudenhoven I., Lennartz J. (2018). Good and Bad Sides of Self-Compassion: A Face Validity Check of the Self-Compassion Scale and an Investigation of its Relations to Coping and Emotional Symptoms in Non-Clinical Adolescents. J. Child Fam. Stud..

[25] Muris P., Otgaar H., López A., Kurtic I., van de Laar I. (2021). The (non)Protective Role of Self-Compassion in Internalizing Symptoms: Two Empirical Studies in Adolescents Demonstrating Unwanted Effects of Using the Self-Compassion Scale Total Score. Mindfulness.

[26] Strauss C., Taylor B.L., Gu J., Kuyken W., Baer R., Jones F., Cavanagh K. (2016). What is compassion and how can we measure it? A review of definitions and measures. Clin. Psychol. Rev..

[27] Gu J., Baer R., Cavanagh K., Kuyken W., Strauss C. (2020). Development and Psychometric Properties of the Sussex-Oxford Compassion Scales (SOCS). Assessment.

[28] Gu J., Cavanagh K., Baer R., Strauss C. (2017). An empirical examination of the factor structure of compassion. PLoS ONE.

[29] Kim J., Seo J.-W. (2021). Assessing Compassion in Korean Population: Psychometric Properties of the Korean Version of Sussex-Oxford Compassion Scales. Front. Psychol..

[30] Karakasidou E., Raftopoulou G., Pezirkianidis C., Stalikas A. (2021). Validity, reliability, and factorial structure of the Self-Compassion Scale-Youth version in the Greek population. Psychology.

[31] Kovacs M. (1992). Children’s Depression Inventory.

[32] Saylor C.F., Finch A.J., Spirito A., Bennett B. (1984). The Children’s Depression Inventory: A systematic evaluation of psychometric properties. J. Consult. Clin. Psychol..

[33] Timbremont B., Braet C., Dreessen L. (2004). Assessing Depression in Youth: Relation Between the Children’s Depression Inventory and a Structured Interview. J. Clin. Child Adolesc. Psychol..

[34] Muris P., Simon E., Lijphart H., Bos A., Hale W., Schmeitz K., Albano A.M., International Child and Adolescent Anxiety Assessment Expert Group (ICAAAEG) (2017). The Youth Anxiety Measure for DSM-5 (YAM-5): Development and First Psychometric Evidence of a New Scale for Assessing Anxiety Disorders Symptoms of Children and Adolescents. Child Psychiatry Hum. Dev..

[35] Garcia-Lopez L.-J., Saez-Castillo A.J., Fuentes-Rodriguez G. (2017). Psychometric properties of the Youth Anxiety Measure for DSM-5, Part I (YAM-5-I) in a community sample of Spanish-speaking adolescents. J. Affect. Disord..

[36] Simon E., Bos A., Verboon P., Smeekens S., Muris P. (2017). Psychometric properties of the Youth Anxiety Measure for DSM-5 (YAM-5) in a community sample. Pers. Individ. Differ..

[37] Soltani E., Bazrafshan A., Moghimi E., Hedayati A., Sheikholeslami S. (2020). Psychometric properties of the Persian Version of Youth anxiety measure for DSM-5 (YAM-5) in nonclinical sample. Arch. Psychiatry Psychother..

[38] Field A.P. (2009). Discovering Statistics Using SPSS.

[39] Kotera Y., Llewellyn-Beardsley J., Charles A., Slade M. (2022). Common Humanity as an Under-acknowledged Mechanism for Mental Health Peer Support. Int. J. Ment. Health Addict..

[40] Ewert C., Vater A., Schröder-Abé M. (2021). Self-Compassion and Coping: A Meta-Analysis. Mindfulness.

[41] Smith B.W., Guzman A., Erickson K. (2018). The Unconditional Self-Kindness Scale: Assessing the Ability to Respond with Kindness to Threats to the Self. Mindfulness.

[42] Kirby J.N. (2016). Compassion interventions: The programmes, the evidence, and implications for research and practice. Psychol. Psychother. Theory Res. Pract..

[43] Dreisoerner A., Junker N.M., Dick R. (2021). The relationship among the components of self-compassion: A pilot study using compassionate writing to enhance self-kindness, common humanity, and mindfulness. J. Happiness Stud..

[44] Avison R., McAlpine D.D. (1992). Gender differences in symptoms of depression among adolescents. J. Health Soc. Behav..

[45] Lewinsohn P.M., Gotlib I.H., Lewinsohn M., Seeley J.R., Allen N.B. (1998). Gender differences in anxiety disorders and anxiety symptoms in adolescents. J. Abn. Psychol..

[46] Yarnell L.M., Stafford R.E., Neff K.D., Reilly E.D., Knox M.C., Mullarkey M. (2015). Meta-Analysis of Gender Differences in Self-Compassion. Self Identity.

[47] Gilbert P. (2014). The origins and nature of compassion focused therapy. Br. J. Clin. Psychol..

